# Release of PAHs from reclaimed asphalt mixtures into the water environment after passivation by cold in-place recycling technology

**DOI:** 10.1007/s11356-024-35875-2

**Published:** 2025-01-10

**Authors:** Vilma Jandová, Martina Bucková, Jiří Huzlík, Jan Valentin, Karel Effenberger, Josef Svoboda

**Affiliations:** 1 Transport Research Centre, Líšeňská 33a, 636 00 Brno, Czech Republic; 2 Department of Road Structures, Faculty of Civil Engineering, Czech Technical University in Prague, Thákurova 7, 166 29 Prague 6, Czech Republic

**Keywords:** Reclaimed asphalt mixture, Asphalt specimens, 16 EPA PAHs, Leaching, Water contamination, PAH passivation

## Abstract

The paper deals with an analysis of the amount of 16 polycyclic aromatic hydrocarbons (PAHs (Polycyclic aromatic hydrocarbons—16 defined by US EPA.)) released from reclaimed asphalt mixtures used in base layers of road surfaces and in binder layers in road construction using cold in-place recycling. For the ten samples tested, the sum of 16 PAHs was determined directly for the crushed asphalt mixture and for its 24-h leachate. The crushed asphalt samples were used to make cylindrical asphalt specimens of the cold recycling mixture. The asphalt specimens were prepared to simulate as realistically as possible the use of the mixtures in road reconstructions implementing the cold in-place recycling technology, which should ensure the passivation of PAHs. The asphalt specimens were subjected to a dynamic leaching test under laboratory conditions with regular replacement of the leaching liquid. The results showed that the concentration of PAHs released into the water environment from the test specimens (passivated material) was approximately 40–50% lower than the amount of PAHs released from the asphalt mixtures in the 24-h leaching test. When compared to the input PAH concentrations in the asphalt mixtures (36–1323 mg.kg^−1^), on average, only 0.15% of PAHs is released from the passivated material. Dynamic leaching test has shown that the wrapping of the original asphalt mixture with a new binder and its subsequent compaction leads to the preservation of PAHs in the original material at such a level that their total concentrations are reduced by two orders of magnitude.

## Introduction

Reconstructions and repairs of road infrastructure are nowadays a way to source asphalt materials that are often being reused, especially in the reconstruction of existing roads. The use of these materials is desirable as it promotes a circular economy, which leads to saving natural resources and consequently saving money that would have been spent on the disposal of these materials in waste disposal sites (Agrela et al. 2021; Bamigboye et al. 2021; Huang et al. 2009). However, it is important to note that these materials contain varying concentrations of PAHs and other pollutants such as metals (Azadgoleh et al. 2024; Mijic et al. 2020; Yang et al. 2020). PAHs are one of many groups of hydrocarbon compounds, and some types of these compounds are classified as harmful or hazardous substances. As they are an integral part of petroleum products and tars used in the past, they are commonly found in older asphalt materials (Muñoz et al. 2022; Xiu et al. 2020). Some PAHs falling under the category of hazardous substances have demonstrable negative effects on the environment and human health; among these substances, benzo(a)pyrene is a known severe human carcinogen (IARC 2012).

In order to allow the reuse of asphalt materials in road construction, despite the risk of higher PAH concentrations, legislation was adopted in the Czech Republic (MoE of the CZ 2019) which provides for the conditions and procedures under which this material can be used. The main indicator used for the evaluation of reclaimed asphalt mixtures is the amount of the sum of 16 defined PAHs (identified as primary pollutants) and the concentration of benzo(a)pyrene determined directly in the asphalt mixture (grit). If high concentrations of PAHs are detected, i.e. the sum of 16 PAHs is greater than 25 mg.kg^−1^ dry matter, the material is always primarily viewed as waste when recovered from the asphalt pavement. However, subject to adherence to legislative conditions, even these “contaminated materials” can be used if cold in-place recycling technology is applied, using an asphalt binder in the form of asphalt emulsion or foamed asphalt, alone or in combination with a suitable hydraulic binder present in a smaller proportion than the asphalt binder. These techniques use the process of passivation of the PAH effects, whereby the asphalt film formed by mixing the materials with the asphalt binder “locks” the contaminant so that it is not released into the environment.

The PAH concentration determined directly in the dry matter of the reclaimed asphalt mixtures indicates the level of contamination of the mixture. However, the result does not tell us anything about the actual amount of the contaminant released into the environment surrounding the road, where rainfall and running water can lead to leaching of the contaminant into the soil layers and the water environment (Azadgoleh et al. 2022; Licbinsky et al. 2012; Norin and Strömvaix 2004). Therefore, these results alone cannot be used to determine what impacts the reuse of “contaminated” asphalt mixture may have on the various components of the environment. We can partially determine the amount of PAHs released from the asphalt mixtures into the water environment by carrying out a 24-h leaching test analysing the granular materials with a grain size < 10 mm at a solid-to-liquid phase ratio of 1:10. The results of this test indicate the concentration levels of the PAHs that enter the water environment from the eroded material particles over a 24-h period and can therefore help to partially describe the release of PAHs in asphalt mixtures used on the ground surface or in unbound road layers in the granular state (Brandt and De Groot 2001). However, even this approach is inadequate to analyse asphalt mixtures contained in bound road layers, where the reclaimed material is encased in a new binder and compacted, i.e. passivated. In such a case, it is appropriate to use a dynamic leaching test using test specimens formed from the original crushed asphalt mixture and the binder. The results of the dynamic leaching test indicate the actual amount of PAHs released into the water environment after passivation of the contaminated asphalt mixtures and therefore indicate the effectiveness of passivation (Fathollahi et al. 2022; Paulus et al. 2016).

This paper discusses the release of PAHs from ten asphalt mixture samples of different concentrations into the water environment before and after the PAH passivation. First, the samples were subjected to a 24-h leaching test to assess the amount of contaminant released directly from the asphalt grit into the water environment. Subsequently, test asphalt specimens were created from the original reclaimed asphalt mixtures using passivation technology. The test specimens were then subjected to a dynamic leaching test according to the methodology “Environmental Requirements for the Use of Recycled Asphalt Mixtures in Road Construction” (Licbinsky et al. 2018), which is based on the leaching of monolithic specimens in predefined time with periodic replacement of the leaching liquid for each time period. The leaching process essentially simulates the exposure of the passivated asphalt material to external influences under laboratory conditions. The test is designed to be as close as possible to the real-world conditions. The amount of PAHs released from each leaching process was compared, and the extent of potential environmental contamination from the reuse of PAH-contaminated asphalt mixtures was evaluated.

The dynamic leaching test is time and financially demanding, which leads to its limited use in practice. The aim of the project was to modify this test so that it could be implemented in a simplified mode. The analysis for the first three stages of leaching was therefore replaced by a mathematical calculation, which was set up in such a way that important information needed to evaluate the test would not be lost. It is possible to believe that this “shortened” procedure could contribute to the more frequent use of this test in practice. Another goal of the project was to compare the results of the 24-h aqueous leaching of the crushed material with the results of the dynamic leaching test, which better simulates the actual situation when reclaimed asphalt mixtures are used.

## Material and methods

Ten samples of reclaimed asphalt mixtures (A1–A10) were subjected to chemical analyses to determine the input amount of the sum of 16 PAHs before the leaching tests. The input analysis was also performed on the binder (P1), which was subsequently used to produce leaching monolithic specimens and passivation of PAHs. In addition, a 24-h leachate was prepared for the input mixtures and the new binder to determine the amount of PAHs released into the water environment. The reclaimed asphalt mixtures were subsequently used to produce test asphalt specimens based on the general requirements of a regulation of the Ministry of Transport (Technical Regulation 2009), which were subjected to dynamic leaching tests in accordance with the Methodology (Licbinsky et al. 2018) (the test specimens are referred to as M1–M10). In contrast to the standard method, where test cylindrical specimens are created with a diameter of 150 mm and compacted by static pressure, for the purpose of the leaching tests within the present analysis, test specimens with a diameter of 100 mm were used, which were compacted by 2 × 50 blows of an impact compactor according to EN (2020). The test specimens were manufactured according to a recipe containing 93.5% reclaimed asphalt mixture, 3.5% cationic asphalt emulsion, 2% water and 1% CEM II 32.5 cement. The cylindrical specimens with a diameter of 100 mm were produced by impact compaction, applying 2 × 50 blows. In all the leachates obtained from the 24-h tests and dynamic leaching tests, the concentration of the sum of 16 PAHs was determined; these were the 16 PAHs defined by US EPA—naphthalene, acenaphthylene, acenaphthene, fluorene, phenanthrene, anthracene, fluoranthene, pyrene, benz[a]anthracene, chrysene, benzo[b]fluoranthene, benzo[k]fluoranthene, benzo[a]pyrene, indeno[1,2,3-cd]pyrene, dibenz[a,h]anthracene and benzo[ghi]perylene. The results of analyses of individual PAH determination methods were compared with each other using the statistical software QC.Expert v. 3.3 (TryloByte Statistical Software, Czechia).

In the dynamic leaching test, the pH of the aqueous leachates was also monitored. Leaching was carried out using ultrapure water with an average pH value of 6. Ultrapure water was used to maintain its quality during the long-term leaching test and also because its pH value falls within the pH range of rainwater, which in the Czech Republic is in the range of 4.4–6.5.

**Fig. 1:**
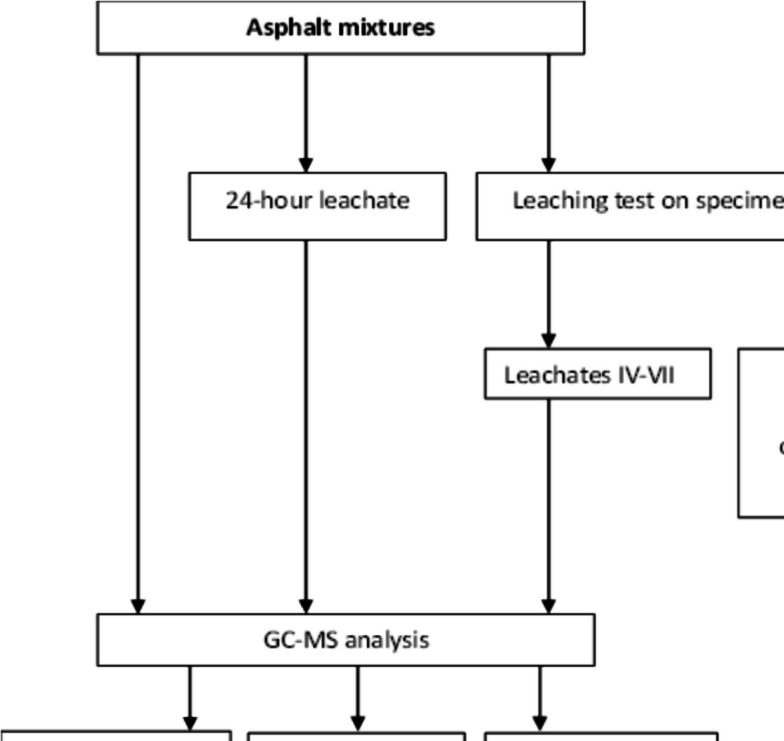
Scheme of work progress within the study

The activities performed in the study are presented in Fig. 1.

### Chemical analysis of input asphalt mixtures

For all ten asphalt mixtures and the binder used for the preparation of the leaching test specimens, the dry matter content of these samples was first determined according to ISO 11465 – Soil quality – Determination of dry matter content on a mass basis – Gravimetric method (ISO 1998). Next, the concentrations of the sum of 16 PAHs in the samples were determined according to an accredited standard laboratory procedure and CSN EN 17503 – Soil, sludge, treated biowaste and waste – Determination of polycyclic aromatic hydrocarbons (PAHs) by gas chromatography (GC) and high-performance liquid chromatography (HPLC) (EN 2022). Samples were pretreated using cryogenic crushing on a jaw crusher (BB 50, Retsch, Germany) and sieved (sieve shaker AS200 control, Retsch, Germany) to a grain size < 1 mm. Afterwards, they were extracted by the Randall extraction method (SER 158, VELP Scientifica, Italy), purified on a glass column filled with silica gel and concentrated. Analysis was performed by gas chromatography (GC) with mass detection (MS) using a GCMS device Agilent 8860-GC/5977B-MSD/7693A-AI (Agilent, USA) (Bozek et al. 2016). To distribute PAHs according to their affinity for the stationary phase, we employed a J&W Agilent Select PAH GC fused silica capillary columns (Agilent Technologies, USA) 30 m length with an internal diameter of 250 µm and the stationary phase with a film thickness of 0.25 µm, which gives a more detailed PAH distribution than do existing commonly used columns. H_2_−6.0-quality hydrogen (SIAD, Italy) was employed as carrier gas with a constant flow of 1.3 ml.min^−1^. The following temperature program with a run time of 27 min was used to determine the PAHs: 80 °C for 0 min, 25 °C.min^−1^ to 200 °C for 0 min, 10 °C.min^−1^ to 220 °C for 4 min, 10 °C.min^−1^ to 325 °C for 0 min, 50 °C.min^−1^ to 350 °C for 5.2 min. Injection was pulsed splitless with 20 psi until 0.75 min. The selected ion monitoring (SIM) method was used to quantitatively assess the PAH concentrations. Calibration was performed using standard calibration solutions PAH-MIX 63, PAH-MIX 183 (Dr. Ehrenstorfer GmbH, Germany) and the PAH Interferences Standard (Restek, USA) in the range of 1–3000 ng.mL^−1^.

### Leachates of the input asphalt mixtures and leaching test specimens

**Fig. 2:**
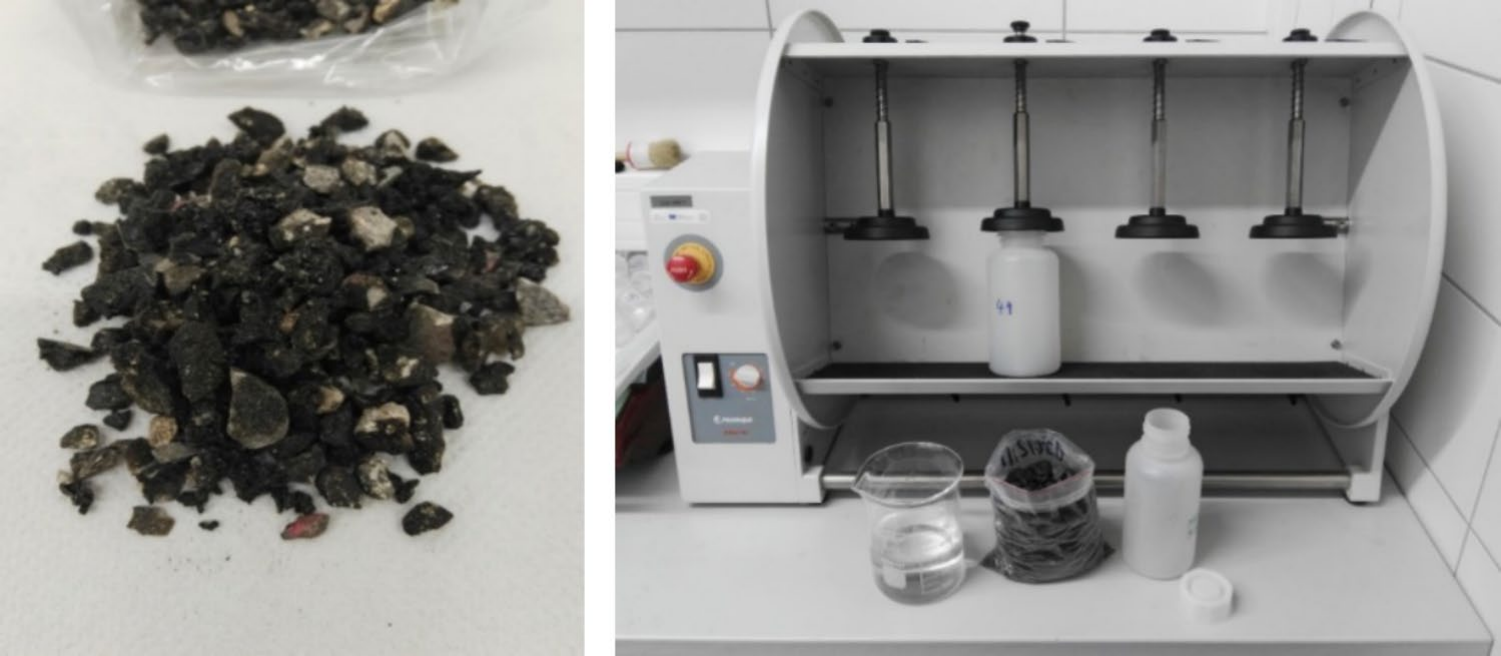
Input asphalt mixture, preparation of 24-h leachate

After determining the concentration of the 16 PAHs in the input reclaimed asphalt mixtures (Fig. 2) and in the asphalt binder used for the preparation of the cylindrical asphalt specimens, the concentrations of this parameter were also determined in the 24-h leachates (± 0.5 h) according to EN 12457–4 – Characterisation of waste – Leaching – Compliance test for leaching of granular waste materials and sludges. Part 4, One stage batch test at a liquid-to-solid ratio of 10 L.kg^−1^ for materials with particle size below 10 mm (EN 2003). Prior to leaching, samples of the input asphalt mixtures were pretreated using cryogenic grinding on a jaw crusher (BB 50, Retsch, Germany) and sieving (AS200 control, Retsch, Germany) to a grain size < 10 mm. The sample amount to be leached and the volume of leaching liquid were accurately determined based on the dry matter of the sample. A sample weight of ± 50 g was placed into the prepared PTFE sampler and subsequently flooded with ± 500 mL of ultrapure water and shaken for 24 h on a rotary shaker (Reax 20, Heidolph, Germany) in overhead swirling action mode at a rotation speed of 5–10 rpm (Fig. 1). After completion of the leaching process, the bottles with leachate were always left in a resting state for 15 ± 5 min to allow the particles suspended in the leachate to settle. Subsequently, the entire volume of leachate was centrifuged (Universal 320 R, Hettich, Germany) for 20 min at 4000 rpm.

**Fig. 3:**
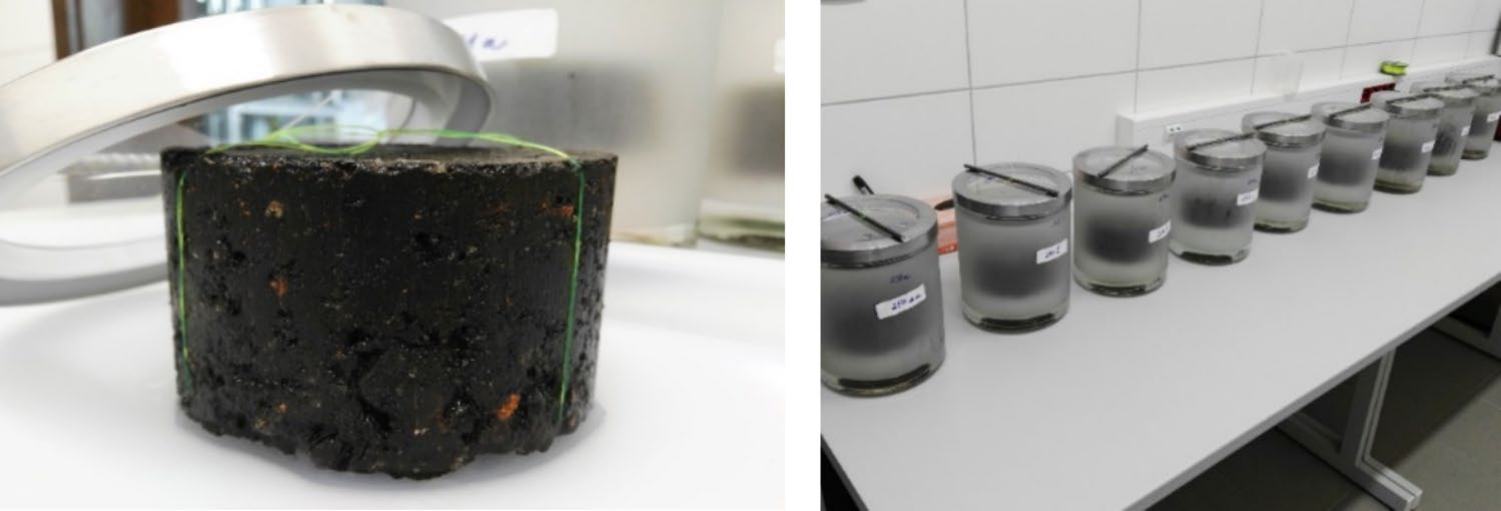
Cylindrical test specimen prepared from the original asphalt mixture, leaching test

Leaching of the test cylindrical specimens was carried out by a dynamic leaching test with periodic replacement of the leaching liquid according to the procedure of the Methodology (Licbinsky et al. 2018). Three test cylindrical specimens with a diameter of 100 ± 1 mm and a maximum height of 50 mm were made from each sample of the input asphalt mixture according to the abovementioned recipe. Glass tanks with a volume of 3.5 L were prepared for the leaching test (Fig. 3). The size of the leaching tank was determined based on the surface area of the leaching specimens, their volume and the volume of the leaching liquid for all test specimens. For the leaching test, a minimum distance of 2 cm between the test specimen, the walls and the level of the leaching liquid in the tank was observed. Each test specimen was first thoroughly cleaned of dust particles and suspended in the leaching tank on a PTFE line tether.

The test specimen was placed in the leaching tank and flooded with a precise volume of ultrapure water calculated according to the surface area of the respective test specimen and the given liquid-to-solid leaching ratio, which is set by the Methodology at 8 ± 0.1 mL.cm^−2^ (Licbinsky et al. 2018). A total of seven leaching time stages were then used for the leaching of each specimen (Table 1).

**Table 1:** Design of the dynamic leaching test

Stage	Leaching time	Analysis
I	6 h	-
II	18 h	-
II	1 day and 6 h	-
IV	1 day and 18 h	Yes
V	5 days	Yes
VI	7 days	Yes
VII	20 days	Yes

After reaching the appropriate time stage, the test cylindrical specimens were always removed from the leaching tank, the leachates were poured into prepared glass samplers and the specimens were returned to the leaching tank which was refilled with an adequate volume of ultrapure water. This procedure was repeated until the last stage of the leaching test. The entire leaching process was carried out in an air-conditioned room at a constant temperature of 20 °C and at dark. The total duration of the dynamic leaching test was 36 days.

### Chemical analysis of the leachates

All leachates, obtained from both the 24-h leaching test and dynamic leaching test, stages IV–VII, were subjected to determination of the content of the 16 PAHs. Sample preparation and determination were performed according to the laboratory’s standard operating procedure and according to ISO 28540 – Water quality – Determination of 16 polycyclic aromatic hydrocarbons in water – Method using gas chromatography with mass spectrometric detection (GC–MS) (ISO 2012). A volume of 500 mL of leachate was extracted in a separatory funnel with a volume of 1000 mL with extraction solvent (3 × 30 mL dichloromethane). After drying the leachate with 2 g of sodium sulphate, anhydrous, it was concentrated to a volume of 1 mL (TurboVap, Biotage, Sweden) and purified on a glass column packed with silica gel. Analysis was performed using a GCMS device Agilent 7890B Series/Agilent 7010 Quadrupole MS/MS (Agilent, USA) according to the procedure described above. For all aqueous leachates of the dynamic leaching test, the pH of the leachates was also monitored at individual test stages.

### Calculation of the amount of PAH released from monolithic samples

The resulting sums of the 16 PAHs obtained in the dynamic leaching test were calculated on the basis of the partial results of the leaching stages IV–VII (Table 1), for the individual PAHs for all cylindrical test specimens (M1–M10), in three repetitions (*c*_*i*_). The leaching of stages I–III was carried out, but due to time and financial demands, the leachates were not analysed. Cumulative concentrations of PAHs for stages I–III were calculated as *Rn* using linearised relationships (4) and data measured in leaching stages IV–VII. The determined concentrations of the 16 PAHs in the leachates of the test specimens were the input values for the calculation of the parameter *R* [µg.m^−2^]. *R* expresses the total amount of the respective PAH that passes through a unit surface area of the sample into the leaching liquid in a given leaching test in stages IV–VII (Table 1) (Licbinsky et al. 2018). The amount of the leached PAH that passed through the unit area in each stage was calculated according to the following equation:

1 $${r}_{i}=\frac{10\times {c}_{i}\times \left(k\times \left.A\right)\right.}{A },$$

where

*r*_*i*_: is the amount of the leached PAH that passes through the unit surface area of the sample into the leaching liquid during the corresponding time *i* of the leaching stage [µg.m^−2^],
*c*_*i*_: is the determined concentration of the relevant PAH in stage *i* [µg.L^−1^] as the median of the three concentrations determined in the leachate of the corresponding test specimen,
*k* = 8 ± 2: the ratio of the volume of liquid in contact with the cylindrical test specimen during the leaching test,
*A*: surface of the cylindrical test specimen [cm^2^].

The total amount of the respective PAH that passes through the unit area of the test cylindrical specimen after *i* leaching stages was calculated according to the following equation:

2 $$R= \sum_{j=1}^{i}{r}_{j},$$

where

*R*: is the amount of the leached PAH that passes through the unit surface area of the specimen into the leaching liquid during the corresponding time t_i_ [µg.m^−2^],
*r*_*j*_: see Eq. (1).

The dependence of *R* on the total leaching test time corresponds to the empirical relation

3 $$R=R_{max}\cdot\frac t{P+t},$$

where

*R*: see Eq. (2),
*t*: leaching duration [day],
*R*_*max*_: maximum leachable amount of the pollutant [µg.m^−2^],
*P*: empirical constant (the time it takes to leach half of the leachable PAH) [day].

In the case of omitting the first *n* analyses, Eq. (3) has the form.

4 $$R'+R_n=R_{max}\cdot\frac t{P+t},$$

where

*R’*: modified amount of leached PAH that passes through a unit surface area of the test specimen into the leaching liquid over the time t_i_-t_0_ of the leaching test [µg.m^−2^],

Both empirical parameters were calculated after linearisation in a spreadsheet using a linear regression function, after data transformation. In this case, the following equation was used for the regression:

5 $$Y=Q\cdot X$$

where

*Q*: linear regression coefficient [-],
*X*: independent transformed variable [-],
*Y*: dependent transformed variable [-],

and the following equation for the transformation in the case of omitting the first *n* analyses:

6 $$Y=\frac{{{R}{^\prime}}_{m}}{{R}{^\prime}}-1,$$

7 $$X=\frac{{t}_{m}-{t}_{0} }{t-{t}_{0}}-1,$$

where

*R, t*: see Eq. (3),
*t*_*m*_: total time of the leaching test (Table 1) [day],
*R’*_*m*_: the amount of the leached PAH that passes through a unit surface area of the test specimen into the leaching liquid over the total time *t*_m_-*t*_0_ of the leaching test [µg.m^−2^]
*t*_*0*_: total leaching time of the last unanalysed leachate.

The regression coefficient *Q* was then used to calculate the parameters *R*_max_, *P* and *R*_*n*_ according to the following equations:

8 $$Rmax=\frac{{R'}_m}{1-Q}\cdot\frac{t_m-t_0}{t_m-\frac{t_0}Q},$$

9 $$P=\frac{Q\cdot t_m-t_0}{1-Q},$$

10 $$R_n=\frac{{R'}_m\cdot t_0}{Q\cdot t_m-t_0},$$

where

*R’*_*m*_*, t*_*m*_: see Eqs. (6) and (7),
*Q*: see Eq. (5).

The total amount of PAHs (the sum of 16 PAHs) released from the cylindrical test specimen was calculated as the sum of the calculated *R*_max_ values for the 16 individual PAHs. *R*_max_ expresses the maximum possible amount of the respective PAH released from the test cylindrical specimen over an infinite time.

## Results and discussions

The concentrations of the sum of 16 PAHs ranging from 36 to 1323 mg.kg^−1^ were determined for ten samples of reclaimed asphalt mixtures (Table 2). Similar concentrations ranging from units to thousands were also found by Su et al. (2019) in different types of asphalt materials—shingles of different ages and reclaimed asphalt materials from road pavements. In addition, along with the analyses of the input concentrations of the mixtures, the asphalt binder P1 obtained by evaporating water from the asphalt emulsion was also analysed and subsequently used to produce test specimens for the dynamic leaching tests. The results of the input mixtures clearly show that mixtures containing tens, hundreds and thousands of mg.kg^−1^ of the sum PAHs were selected for the leaching tests. The concentration of the sum of PAHs determined in the P1 binder was 1.21 mg.kg^−1^ dry matter. This is a very low concentration that essentially could not affect the resulting PAH concentrations in the leaching test specimens. The sum of 15 PAH concentrations in the bituminous (asphalt) binder ranging from 0.1 to 3 mg.kg^−1^ is also described by Brandt and De Groot (2001) and Legret et al. (2005).

**Table 2:** Input concentrations in the asphalt mixtures and the binder

Identification of the asphalt mixture sample	Concentration of the sum of PAH [mg.kg^−1^ dry matter]
A1	36
A2	94
A3	111
A4	230
A5	367
A6	508
A7	925
A8	1032
A9	1271
A10	1323
P1	1.21

The input asphalt mixtures and the P1 binder were further subjected to 24-h leaching tests. The values of the sum of PAHs in the leachates ranged from 0.08 to 0.69 mg.L^−1^, and the concentration in the binder was determined to be 0.005 mg.L^−1^ (Table 3). The amount of the sum of six PAH congeners (benzo(a)pyrene, benzo(b)fluoranthene, benzo(ghi)perylene, benzo(k)fluoranthene, fluoranthene and indeno(1,2,3-cd)pyrene) ranging from 0.013 to 0.120 mg.L^−1^ in 24-h leaching test of reclaimed asphalt material was determined by Legret et al. (2005). In order to be able to compare the amount of the sum of PAHs in the leachate with the original concentrations of the sum of PAHs in the input mixtures, the resulting concentrations in mg.L^−1^ determined in the leaching test were converted to the amount of sample weight of the respective asphalt mixture for the leaching test. The result of the sum of PAHs in mg.kg^−1^ dry matter therefore indicates the amount of the sum of PAHs released to the water environment in mg per kilogram of the respective asphalt mixture (Table 3).

**Table 3:** Concentrations of the sum of PAHs determined in 24-h leachates for individual asphalt mixture samples and comparison with input concentrations

Sample designation	Concentration of the sum of PAHs [mg.kg^−1^ dry matter]	Concentration of the sum of PAHs in 24-h leachates [mg.L^−1^]	Concentration of the sum of PAHs in 24-h leachates [mg.kg^−1^ dry matter]	Proportion of the leachate concentration to input concentration [%]
A1	36	0.039	0.39	1.083
A2	94	0.032	0.32	0.340
A3	111	0.037	0.37	0.333
A4	230	0.041	0.41	0.178
A5	367	0.039	0.39	0.106
A6	508	0.142	1.42	0.280
A7	925	0.239	2.38	0.257
A8	1032	0.198	1.98	0.192
A9	1271	0.330	3.29	0.259
A10	1323	0.349	3.48	0.263
P1	1.21	0.003	0.03	2.479

The concentrations of the sum of PAHs in the 24-h leachates ranged from tenths to units of mg.kg^−1^ dry matter, compared to concentrations in the input materials that ranged from tens to thousands of mg.kg^−1^ dry matter. The leaching test results confirmed that smaller amounts of PAHs were released from samples A1–A5, i.e. the mixtures with input concentrations of no more than 367 mg.kg^−1^. The resulting concentrations were around 0.5 mg.kg^−1^ dry matter. Licbinsky et al. (2012) reported a concentration of the sum of 16 PAHs in leachate of 0.0183 mg.L^−1^ at a leaching time of 60 days and for a leaching test carried out under real-world conditions at a storage site of an uncompacted asphalt material during normal rainfall periods, with an input PAH concentration of about 40 mg.kg^−1^. For asphalt mixtures with input PAH concentrations ranging from 508 to 1323 mg.kg^−1^ dry matter, the amount of the sum of PAHs released into the water environment over a 24-h period was in the range of units of mg.kg^−1^ dry matter. These were samples A6 to A10, showing concentrations in the leachate ranging from 1.42 to 3.48 mg.kg^−1^ dry matter. The resulting PAH concentrations in the 24-h leachates did not confirm that as the concentrations of the sum of PAHs in the input asphalt mixture increases, the concentration in the leachates increases in direct proportion. The variation in the PAH concentration values in the leachates compared to the input values can be explained by the variability in the surface areas of the aggregate and the original binder of the input materials during the 24-h leaching test. However, it can be concluded that less than 0.5% of the sum of PAHs determined in the input materials leached into the water in 24 h. Specifically, for the ten samples examined, the average value was 0.33%. In the case of the asphalt binder, approximately 2.5% of the sum of PAHs was released into water in 24 h; this should not significantly affect the resulting PAH concentrations in the dynamic leaching test where the binder was used to prepare the test specimens, given the low input concentration of PAH in the binder (1.21 mg.kg^−1^ dry matter). More PAHs were released from the binder into the water environment probably due to the higher fineness of the material used in the leaching test.

**Fig. 4:**
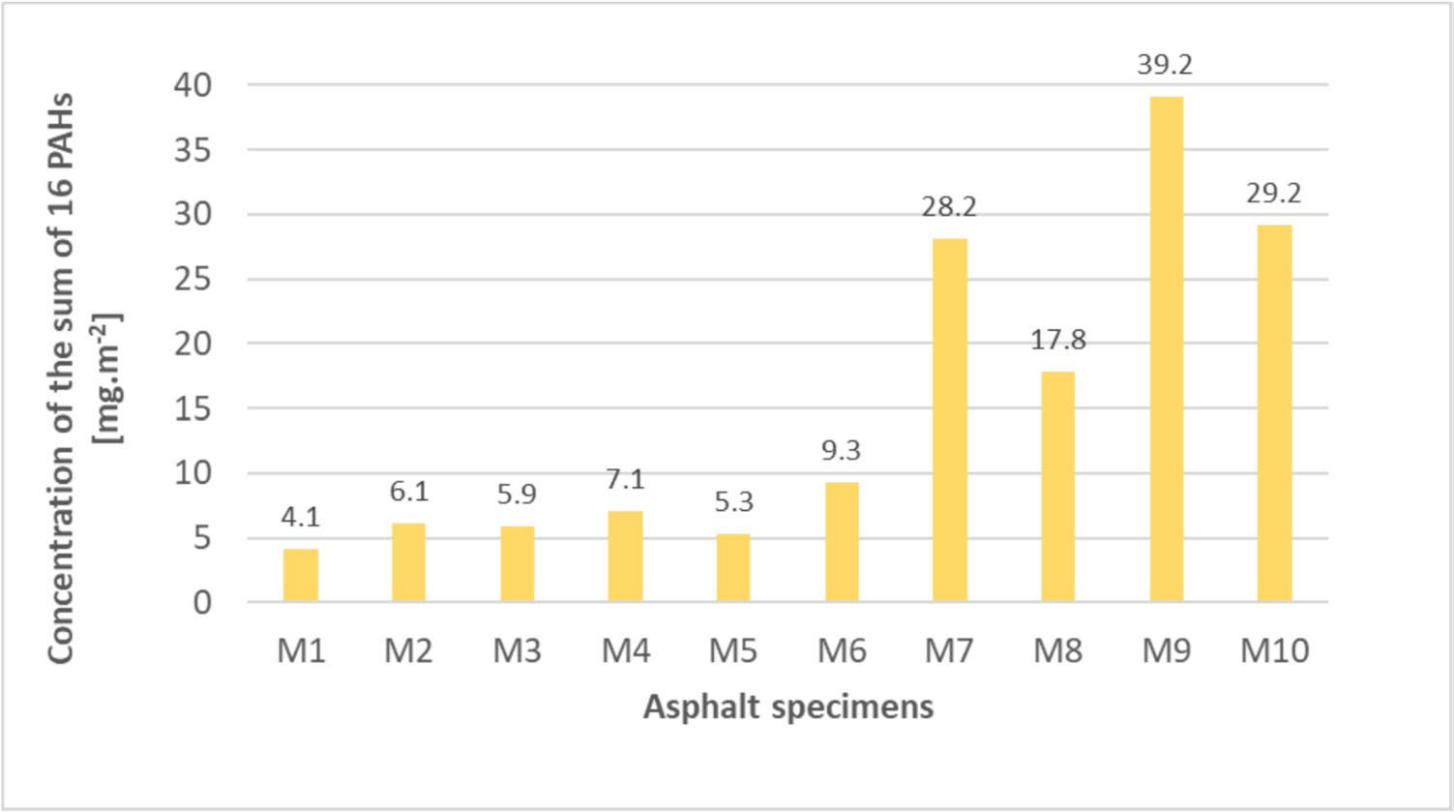
Maximum possible concentrations of the sum of PAHs released from the test specimens into the water environment

The results of the sum of PAHs of the dynamic leaching tests were processed according to the calculation procedure specified in the Methodology (Licbinsky et al. 2018); see Eqs. (1)–(8). The values of the sum of PAHs in the leachates ranged from 0.05 to 0.49 mg.L^−1^. The resulting values for the individual test specimens are primarily related to the area [mg.m^−2^], to give an idea of the amount of the sum of PAHs that can be released when asphalt mixtures are used in real-world conditions, i.e. relative to the road area. This is the maximum possible amount of PAHs released from the surface of the test specimens over an infinite period when PAH leaching from the surface of the pavement no longer occurs. The results are shown in Fig. 4.

The highest amount of the leached sum of PAHs was determined in samples of test specimens that contained the highest input concentrations during their production (samples M7–M10), when the PAH concentrations ranged around 1000 mg.kg^−1^ and even exceeding this value (925–1323 mg.kg^−1^). On the other hand, samples with lower input concentrations (M1–M6) showed lower values, with mixtures having input PAH values between 36 and 508 mg.kg^−1^. Overall, it can be concluded from the results that when the input concentrations of PAH in asphalt mixtures reach maximum values of around 500 mg.kg^−1^ and these mixtures are coated with a new binder of the composition mentioned above and compacted, the amount of the sum of PAHs not exceeding 10 mg.m^−2^ of road surface area is released into the water environment from the surface of these reused asphalt mixtures. If the input concentrations of the asphalt mixtures are in range around 1000 mg.kg^−1^ or more, than the amount of the sum of PAHs of several tens of mg per m^2^ of surface area is released into the water environment from the reused materials. The release of PAHs into the water environment is also influenced by the porosity of the material, which affects the amount of leachate that penetrates the test specimen. Fathollahi et al. (2022) carried out a leaching test of test specimens prepared from aggregate, bitumen and shredded rubber (waste tyres) in different proportions of the different components. The dynamic leaching test was carried out at eight fixed time intervals with the replacement of the leaching liquid. The input concentrations of the sum of PAHs of the granular asphalt mixtures ranged from 9 to 11 mg.kg^−1^. The sum of PAHs in the dynamic leaching test was very low ranging between 0.005 and 0.007 mg.m^−2^. The low concentrations are probably caused by the different production technologies of the test specimens and the materials used, which created a compact surface of the specimens where the leaching liquid did not penetrate to a greater depth of the test specimen material.

The pH values monitored for all stages of the leaching test (I–VII) gradually increased, from an average value of 6.7 for stage I leachates to an average value of 9.7 for stage VII leachates. A significant increase in pH occurred in the fifth leaching step, when the value increased by an average of 3 units (pH 9.6) compared to the input value (pH 6). The reason is a significantly longer leaching period (5 days) than in previous time stages. The pH value increases with increasing leaching time.

**Fig. 5:**
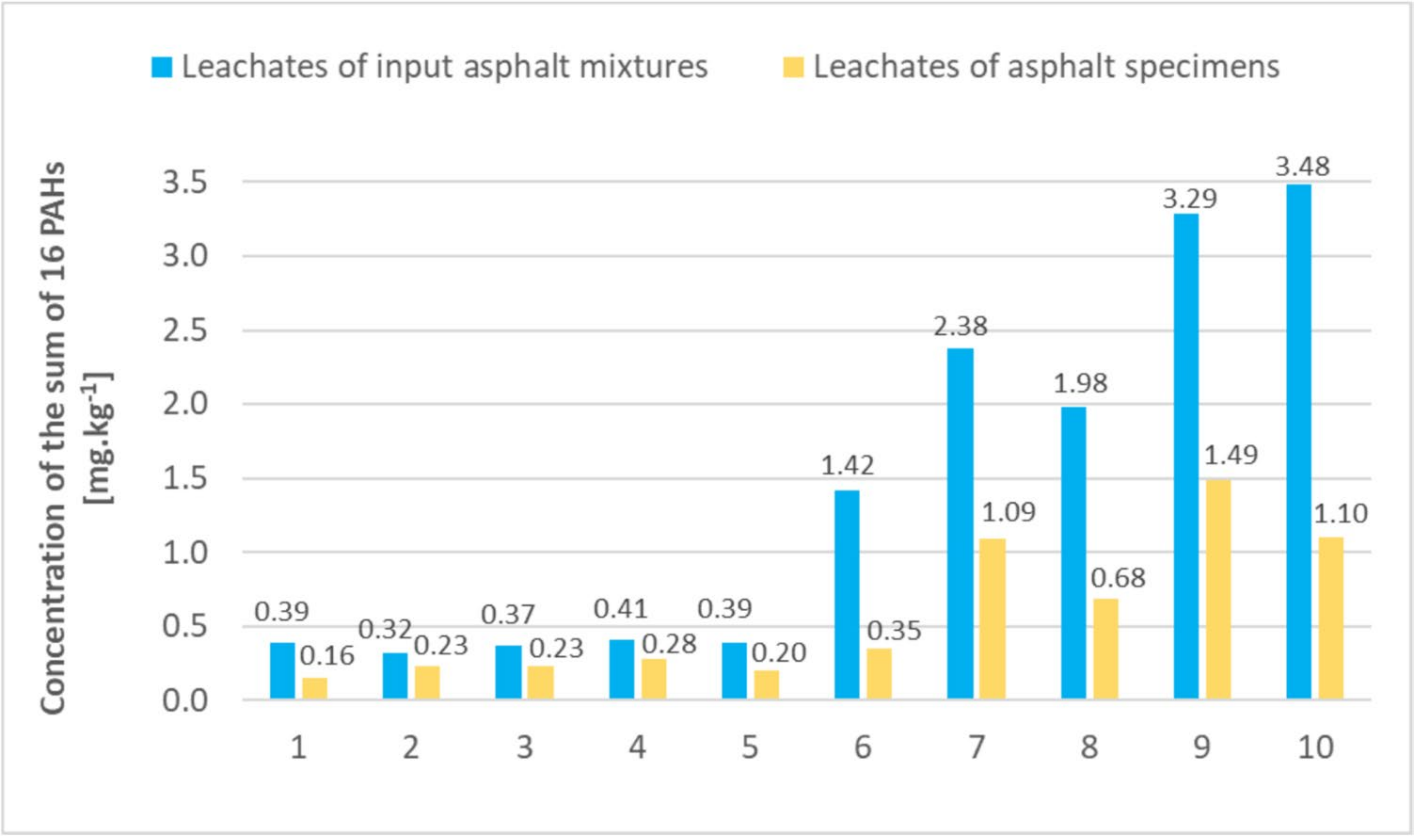
Comparison of the sum of PAH concentrations in the 24-h and dynamic leaching test leachates

To compare the resulting sum of PAH concentrations from the dynamic leaching test with the data obtained from the 24-h leaching test, the resulting concentrations were again converted from the amount of released PAHs relative to the surface area to the amount of the sum of PAHs relative to the mass of the leaching specimens, i.e. mg.kg^−1^. Thus, the resulting concentration of the sum of PAHs in mg.kg^−1^ is indicative of the amount of PAHs released into the water environment in mg per kg of test specimen. A comparison of the results of the two leaching tests is given in Fig. 5.

The sum of PAH concentrations determined in the 24-h leaching test was higher for all ten mixture samples than the concentrations determined in the dynamic leaching test of the cylindrical test specimens. The concentration trends for each sample were similar for both leaching tests. For both leaching tests, higher amounts of the sum of PAHs were consistently determined for the leachates of samples with input values of the sum of PAHs ranging from 927 to 1323 mg.kg^−1^. Conversely, lower concentrations were determined for samples where the sum of PAHs in the input mixtures did not exceed 508 mg.kg^−1^. The test specimens of samples M1 to M6 released on average 50% less PAHs into the water environment, while samples M7 to M10 released on average 40% less PAHs than was the case for the input mixtures over 24 h. A similar result was also achieved by Brandt and De Groot (2001) when comparing the results of asphalt testing with a 30-h leaching test at a solid-to-liquid ratio of 1:10 and leaching tests with periodically replacing leaching liquid at intervals of 0.25, 1, 2, 25, 4, 9, 16 and 36 days. The concentrations of the sum of 16 PAHs showed 45% higher values at 24-h leachate compared to the dynamic leaching test. The results of total PAH concentrations for both types of leaching were statistically evaluated. Since these were the results of analysis of the same samples under different conditions, which were correlated with each other (determination coefficient *R*^2^ = 0.9016), a pairwise comparison of the results of the sets was used to compare both types of aqueous leaching. This showed the statistical significance of the differences in PAH concentrations obtained by these methods (*p* = 0.00455).

The results of both leaching tests clearly show that by covering the reclaimed asphalt mixtures with a new asphalt binder and subsequent compaction, PAHs are preserved in the newly formed composite material, which clearly leads to a reduction in the release of this contaminant into the surrounding environment. Fathollahi et al. (2022) arrived at the same conclusion when comparing the sum of 16 PAHs contained in crushed rubber granulate with the amount of the sum of 16 PAHs released into water in a dynamic leaching test of asphalt specimens containing rubber granulate. Azadgoleh et al. (2024) also confirmed in their research that the use of geopolymer in asphalt mixtures effectively reduces the leaching of contaminants into the surrounding environment. Therefore, after passivation of the asphalt mixture, the amount of PAHs released into the water environment over a 24-h period is halved compared to the amount released from the original crushed asphalt mixture. The findings were confirmed by the results of dynamic leaching tests, which showed that an average of 0.15% of the sum of PAHs of the total input concentration in the asphalt mixture samples was released into the water environment from the ten tested specimens. Statistical evaluation of the amount of PAHs using the ANOVA method was performed between the values of the input concentrations and the resulting concentrations of both leaching tests for all samples (24-h leaching, resp. dynamic leaching). The results showed statistical significance between the input values and both aqueous leachates (*p* = 0.00049, resp. *p* = 0.00048).

The subject of the research solution was not the investigation of the structure of the asphalt material or directly the mechanism of leaching of the contaminant into the water environment. The research was focused on the release of PAHs into water from various contaminated asphalt mixtures before and after they were coated with a new binder. The research results bring new knowledge regarding the possibility of reusing asphalt materials contaminated with PAHs, which would have been considered waste in the past, for the repair of existing roads.

## Conclusion

The aim of the study was to determine the amount of PAHs that can be released from differently contaminated asphalt mixtures into water. Ten samples of asphalt materials were selected, for which the total amount of 16 PAHs was determined. The concentrations ranged from 36 to 1323 mg.kg^−1^ of dry matter. The samples were subjected to 24 h of aqueous leaching. Furthermore, cylindrical test bodies were made from them, which were subjected to a dynamic leaching test. The results of all parts of the research were subsequently compared, and the rate of PAHs leaching into the aquatic environment was evaluated.

The results of the experiment showed that during 24 h of leaching of the asphalt mixture in water, approximately one-half of 1% of PAHs from the specified input concentrations is released into the water environment. The amount of the sum of PAHs in the asphalt binder was very low in both the input mixture and the 24-h aqueous leachate of the binder. Therefore, it was concluded that the binder could not have a significant impact on the resulting values of the sum of PAHs for the dynamic leaching test of the test specimens.

The concentrations of the sum of PAHs in the leachates of the cylindrical test specimens were lower than those in the 24-h leachates for all samples. Comparing the results of the two leaching tests (24-h and dynamic tests), it was shown that for the test specimens in which the original reclaimed asphalt mixtures is coated with an asphalt binder and compacted (i.e. a composite mixture is created corresponding to the mixture created in practice by cold in-place recycling technology), approximately 40–50% lower amount of PAHs is released into the water environment than for the 24-h leachate of the original asphalt mixture. If the resulting PAH concentrations obtained in the dynamic leaching are compared to the original input concentrations, on average, only 0.15% of the total input concentration of the sum of PAHs was released into the water environment.

It can be concluded that the dynamic leaching test performed according to the Methodology (Licbinsky et al. 2018) has shown that the coating of the original reclaimed asphalt mixture with a new binder and its subsequent compaction leads to the preservation of PAHs in the original material at such a level that their total concentrations are reduced by two orders of magnitude (from the original values ranging tens, hundreds and thousands of mg.kg^−1^). It should also be noted that the most contaminated reclaimed asphalt materials are usually reused in road construction in the base layers of road pavements (occasionally cold in-place recycling technology is used in the binder layer), where the direct contact of the mixture with surface water due to rainfall should be limited. Therefore, in real-world conditions, the amounts of PAHs released into the surrounding environment are likely to be even smaller than the results of this experiment have shown.

The dynamic leaching methodology could also be used to verify the passivation of not only PAHs on other types of passivating asphalt binders or cement-based binders. If we wanted to simulate real conditions even better, it would be advisable to modify the method for leaching with a constant stream of flowing water. In this case, the aspect of water movement on the road would also be included in the test. The use of the dynamic leaching method is especially suitable for the state administration and companies that deal with the reconstruction of transport infrastructure. Based on this test, they could be demonstrating the suitability of reusing contaminated asphalt material, which leads to the prevention of waste, conservation of natural resources and preservation of sustainable development.

## Data Availability

The datasets will be available from the corresponding author upon reasonable request.
